# Advanced imaging tools for evaluating cardiac morphological and functional impairment in hypertensive disease

**DOI:** 10.1097/HJH.0000000000002967

**Published:** 2021-08-12

**Authors:** Maria Lembo, Maria Virginia Manzi, Costantino Mancusi, Carmine Morisco, Maria Assunta Elena Rao, Alberto Cuocolo, Raffaele Izzo, Bruno Trimarco

**Affiliations:** Department of Advanced Biomedical Sciences, Federico II University of Naples, Naples, Italy

**Keywords:** 3D-echocardiography, arterial hypertension, cardiac magnetic resonance, computed tomography, speckle tracking echocardiography

## Abstract

Arterial hypertension represents a systemic burden, and it is responsible of various morphological, functional and tissue modifications affecting the heart and the cardiovascular system. Advanced imaging techniques, such as speckle tracking and three-dimensional echocardiography, cardiac magnetic resonance, computed tomography and PET-computed tomography, are able to identify cardiovascular injury at different stages of arterial hypertension, from subclinical alterations and overt organ damage to possible complications related to pressure overload, thus giving a precious contribution for guiding timely and appropriate management and therapy, in order to improve diagnostic accuracy and prevent disease progression. The present review focuses on the peculiarity of different advanced imaging tools to provide information about different and multiple morphological and functional aspects involved in hypertensive cardiovascular injury. This evaluation emphasizes the usefulness of the emerging multiimaging approach for a comprehensive overview of arterial hypertension induced cardiovascular damage.

## INTRODUCTION

Arterial hypertension (AH) represents a relevant cardiovascular risk factor and a systemic burden [1,2]. It has, in fact, an important impact on the development of cardiac and vascular events, predisposing to heart failure, acute coronary syndromes, peripheral artery disease and stroke [3,4]. AH affects the entire body, thus leading to target organ damage in multiple districts [5].

Early diagnosis of cardiac modifications induced by pressure overload and arterial disease induced by increased arterial stiffness, together with the identification of subclinical organ damage have become crucial for a timely and adequate treatment, aiming at not only the avoidance of irreparable organ damage but also trying to confine injury progression [6].

Beyond standard echo-Doppler echocardiography, which remains among the most simple but accurate methods for the investigation of cardiac and vascular remodelling and diastolic dysfunction [1,6,7], advanced imaging techniques are emerging for their sensitive capability of recognizing early myocardial and vascular impairment in many cardiovascular diseases [8–10], including AH [11,12]. Those imaging methods, including speckle tracking and three-dimensional (3D) echocardiography, cardiac MRI, computed tomography (CT) and PET-computed tomography (PET-CT), can help, in different ways, in the detection of subclinical disease and/or in the identification of cardiovascular changes deriving from pressure overload [13–15].

The current review aims at highlighting the usefulness of advanced imaging tools, each with its peculiarity, beyond standard echocardiography, for the identification of subclinical AH-induced organ damage, revealing both myocardial morphological and functional alterations present in the hypertensive setting, even at very early stages.

## TWO-DIMENSIONAL SPECKLE TRACKING ECHOCARDIOGRAPHY

Two-dimensional (2D) speckle tracking echocardiography is a feasible ultrasound tool, providing information about left ventricular deformation in different directions: longitudinal, circumferential and radial and left ventricular twisting. It was demonstrated that all speckle-tracking-derived strains appeared to be altered in patients affected by AH [16]. In particular, global longitudinal strain (GLS), despite being load dependent, is a sensitive parameter for identification of left ventricular subclinical systolic dysfunction, and it is related to the amount of myocardial fibrosis and left ventricular filling pressures [17,18]. In fact, it resulted impaired even in patients at very early stages of AH, independently on alterations in left ventricular geometry and before a reduction in left ventricular ejection fraction, the main used parameter for the evaluation of left ventricular systolic function, also presenting a higher reproducibility and lower interobserver and intraobserver variability than the latter [19,20].

Regional strain provided further information: longitudinal strain impairment mainly involved basal and middle segments, with a relative sparing of apical ones and resulting in higher values of relative regional strain ratio (the ratio between apical and the sum of basal and middle strain) in newly diagnosed hypertensive patients in comparison to controls, thus exacerbating the base-to-apex gradient deformation [21]. This phenomenon found correspondence in myocardial fibrosis by late gadolinium enhancement cardiac magnetic resonance, which is mainly found in left ventricular basal and middle segments in hypertensive patients with concentric left ventricular hypertrophy (LVH) [22].

Left ventricular longitudinal strain resulted progressively more impaired in hypertensive patients presenting left ventricular concentric remodelling, LVH and LVH associated with dilation. Circumferential and radial strains resulted instead compromised in more advanced stages of AH when LVH was already established, being still normal or actually increased as for radial strain and left ventricular torsion in early phases of AH, probably as a compensatory mechanism in response to pressure overload [16,23,24].

Subsequent studies showed the involvement of all the three endo, mid and subepicardial layers of both longitudinal and circumferential strains in AH [25]. At early stages of AH, longitudinal endomyocardial layer seemed to be the most affected by pressure overload [26]. Left ventricular longitudinal and circumferential endocardial and mid-myocardial layer strains progressively decreased from normotensive individuals, across patients with masked AH to patients with sustained AH [27]. Subepicardial layer strain seems to be involved in more advanced stages of AH and it was described as a prognosticator of cardiovascular events [28]. The interrelation and concomitant dysfunction of multiple strains together with the impairment of echo parameters examining different myocardial layers (e.g. the independent association between GLS, quantifying the deformation of longitudinal fibres and midwall fractional shortening evaluating the motion of midwall circumferential fibres), demonstrated the comprehensive left ventricular systolic dysfunctional dynamics in AH [16,29]. Furthermore, longitudinal and circumferential early diastolic strain rates were decreased, while late diastolic strain rates were increased in hypertensive patients, reflecting an impaired left ventricular myocardial relaxation and diastolic dysfunction [27].

In addition, investigating myocardial work components by left ventricular pressure-strain loops derived from speckle tracking echocardiography and GLS, a positive and significant correlation was found between systolic blood pressure and both global work index, representing the total work within the left ventricular pressure-strain area, and global constructive work, indicating the work performed during systolic shortening in addition to negative work during lengthening in isovolumetric relaxation, in a population of heathy individuals [30]. Those correlations were also confirmed in the hypertensive setting, where higher values of global work index and global constructive work values were highlighted in comparison to controls, in particular in patients with eccentric and concentric LVH [31]. Wasted work, which represents the work performed during segmental shortening when the aortic valve is closed in isovolumic relaxation or the work performed during systolic segmental lengthening, was also demonstrated to be higher in hypertensive patients. In addition, global efficiency, which is computed as the ratio between constructive work and wasted work of all left ventricular segments, was demonstrated to be preserved in early stages of hypertension, as higher wasted work is balanced by a higher constructive work, whereas this balance is lost in advanced stage when left ventricular dilation is established [32,33]. In addition, myocardial work index and constructive work were significantly increased in patients with uncontrolled and resistant AH in comparison to well controlled hypertensive patients and normotensive individuals. Myocardial work index was also associated with functional capacity alteration in terms of oxygen consumption [34]. In hypertensive patients, work index was positively correlated with SBP and segmental differences in work index were demonstrated, affecting especially basal segments and in particular basal septum in patients with LVH, emphasizing again the base-to-apex gradient [35].

Moreover, GLS was demonstrated to be a potential good predictor of major adverse cardiovascular events in a population of asymptomatic patients affected by hypertensive heart disease, when incorporated in a risk score, also including age more than 70 years, concentric LVH and atrial fibrillation [36].

GLS stands out among other strain types not only because its impairment is detectable even at very early stages of AH, but for its high reproducibility and as it is easily performed on board the echo machine, whereas the assessment of the other strains requires additional software.

Even if a small variability among vendors is still present, usefulness and simple approach of left ventricular GLS is considered so remarkable in different pathological conditions, including AH, that it has been considered worthy to be incorporated into the standardization of the echo report [37].

Right ventricle could also be affected by AH, as both ventricles share the interventricular septum, myocardial fibres and pericardium and this could determine ventricular interdependence. Indeed, right ventricular systolic dysfunction was demonstrated to be a remarkable prognosticator of heart failure related to AH [38]. 2D strain imaging revealed right ventricular longitudinal mechanics’ dysfunction in early systemic hypertension by an impairment in peak systolic strain and early diastolic strain rate [39]. Both right ventricular GLS and longitudinal deformation of right ventricular free-wall resulted impaired in hypertensive patients, in particular in untreated and suboptimally treated patients, and longitudinal dysfunction was more evident in the subendocardial than in the mid-myocardial right ventricular wall layer [40,41].

Speckle tracking assessment found its utility also for the evaluation of left atrial compliance.

Apart from the left and right ventricle, left atrium is another cardiac chamber being frequently affected by pressure overload. Before the development of left atrial dilation, impairment in left atrial longitudinal strain was detected in patients with suboptimal blood pressure control and AH, independently on left ventricular longitudinal dysfunction [42–44]. Left atrial peak longitudinal strain, measured at the end of the reservoir phase and left atrial peak contraction strain during left atrial systole, resulted altered even before LVH. Left atrial longitudinal strain was considered a surrogate of left ventricular diastolic dysfunction, being significantly correlated with E/e’ ratio and increased left ventricular filling pressures [45,46].

Moreover, in hypertensive patients with concomitant paroxysmal atrial fibrillation, speckle tracking-derived left atrial reservoir, conduit and pump function were all early impaired [47].

Even if not as widely validated as for left ventricular and left atrial assessment, speckle tracking echo was also applied on vascular districts [48]. In particular, circumferential strain analysed in common carotid arteries was demonstrated to be significantly correlated with carotid intimal-media thickness and arterial stiffness in a population including patients with several cardiovascular risk factors and a high prevalence of AH [49].

**FIGURE 1:**
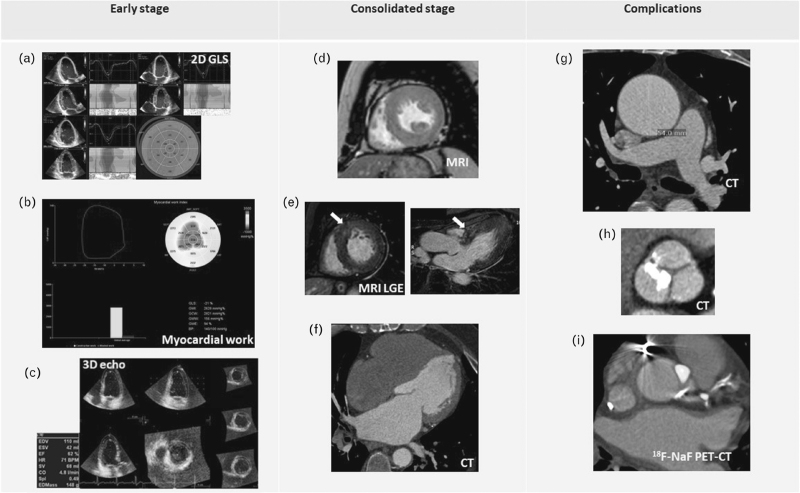
Example showing alterations provided by different advanced imaging tools at early stage, consolidated stage of arterial hypertension and complications. At early stages: (a) 2D GLS and regional strain impairment with predominant involvement of basal and middle longitudinal strain, (b) Myocardial work components alteration in hypertensive patients, (c) LV concentric geometry with increased LVM/EDV ratio detectable at 3D-echocardiography. At consolidated stage: (d) LVH evaluated by MRI, E) LGE nonischemic intramyocardial pattern (white arrow) of the basal anterior septum (left: basal short axis view, right: three-chamber view) in a hypertensive patient with LVH, (f) contrast-enhanced CT showing LA dilation. Complications: (g) Aneurism of the ascending aorta detected by contrast-CT, (h) Aortic valve calcification and stenosis by CT, (i) ^18^F-NaF PET-CT showing uptake within both the descending left coronary artery and the aortic valve. ^18^F-NaF, fluorine-18-sodium fluoride; 2D, two-dimensional; 3D, three-dimensional; CT, computed tomography; EDV, end-diastolic volume; GLS, global longitudinal strain; LA, left atrial; LV, left ventricular; LVH, left ventricular hypertrophy; LVM, left ventricular mass; PET-CT, PET-computed tomography.

Table 1 summarizes the information derived from advanced cardiac imaging techniques for detection of left ventricle (LV), left atrial and vascular damage, according to the possibility to identify morphological and functional abnormalities at an early stage, consolidated stage of AH or possibly diagnosing AH complications (Fig. 1).

**TABLE 1:** Advanced cardiac imaging techniques for detection of left ventricular, left atrial and vascular damage induced by arterial hypertension

Left ventricle			
Tool	Early stage	Consolidated stage	Complications
2D speckle tracking echo	GLS impairment Predominant basal and middle LS involvement Layers’ function impairment, especially longitudinal endomyocardial layer. Myocardial work: higher global work index and global constructive work	GLS, circumferential and radial strains impairment more evident with LV hypertrophy Layers’ function impairment Myocardial work: higher global work index and global constructive work, higher wasted work	
3D echocardiography and 3D speckle tracking	Increased LVM/EDV ratioConcentric remodelling with reduced stroke volumeAll strains impairment: including GLS, global circumferential, radial and area strain.	LV hypertrophy	LV dilation and heart failure
MRI	Impaired LV strains by feature tracking LV diastolic dysfunction	LV hypertrophy Basal and middle segments LGE, with no ischemic pattern LV diffuse and local fibrosis: elevated ECV and native T1 Impaired LV strains by feature tracking LV diastolic dysfunction	LV dilation and heart failure
CT	LV diastolic dysfunction	LV hypertrophy Diffuse fibrosis by CT ECV LV diastolic dysfunction	Coronary artery disease Aortic valve stenosis and Agaston calcium score
PET-CT		Increased LV ^18^F-FDG uptake, with higher inflammation	Increased LV ^18^F-NaF uptake and ^18^F-FDG in aortic valve stenosis Increased LV ^18^F-NaF uptake and ^18^F-FDG in coronary atherosclerosis Rubidium-82 reduced myocardial perfusion reserve

Right ventricle			
Tool	Early stage	Consolidated stage	Complications
2D speckle tracking echo	Impaired RV GLS and free-wall LS	Impaired RV GLS and free-wall LS. Layers’ function impairment, especially longitudinal endomyocardial layer	
3D echocardiography and 3D speckle tracking		3D RV volumes were increased and 3D RV ejection fraction resulted reduced in patients with LV hypertrophy	
MRI		Increased RV mass index, ventricular wall thickness and remodelling index	

Left atrium			
Tool	Early stage	Consolidated stage	Complications
2D speckle tracking echo	LA strain impairment: reduced LA reservoir, conduit and pump function		
3D echocardiography and 3D speckle tracking	LA strain impairment	LA strain impairment and dilation	
MRI	LA strain impairment by feature tracking	LV strain impairment and dilation	
CT		LV dilation	

Vessels			
Tool	Early stage	Consolidated stage	Complications
2D speckle tracking echo	Impaired circumferential vascular strain in carotids		
MRI			Aortic aneurism Carotid plaques
CT			Coronary artery disease Carotid plaques Aortic aneurism Acute aortic syndromesStroke
PET-CT			Increased ^18^F-FDG uptake in carotid atheroma Increased LV ^18^F-NaF uptake in coronary atherosclerosis and carotid plaques

18F-FDG, fluorine-18-fluorodeoxyglycose; 18F-NaF, fluorine-18-sodium fluoride; 2D, two-dimensional; 3D, three-dimensional; CT, computed tomography; ECV, extracellular volume fraction; GLS, global longitudinal strain; LA, left atrial; LS, longitudinal strain; LV, left ventricular; LVM/EDV ratio, left ventricular mass/end-diastolic volume ratio; PET-CT, PET-computed tomography.

## THREE-DIMENSIONAL ECHOCARDIOGRAPHY AND THREE-DIMENSIONAL SPECKLE TRACKING ECHOCARDIOGRAPHY

Real-time 3D echocardiography allows the assessment of left ventricular geometry and in particular the computation of left ventricular mass (LVM) and left ventricular end-diastolic volume (EDV) and end-systolic volume, providing an accuracy that is comparable to MRI, the latter considered the gold standard for this evaluation [50–52]. 3D-echocardiographic technique could represent a good compromise between two-dimensional echocardiography and MRI: it shows indeed advantages over standard 2D-echo, as it does not need geometrical assumptions and could overcome some limitations due to off-axis beam orientation and difficult evaluation of asymmetric LVH; it is also less expensive than MRI [53].

Recently, the use of LVM/EDV ratio, the so-called left ventricular remodelling index, already validated by MRI, has been proposed for the 3D-echocardiographic evaluation of left ventricular geometry in the hypertensive setting [54]. Higher values of LVM/EDV ratio correspond to the increase of left ventricular wall thickness in relation with left ventricular internal cavity size. Elevated values of LVM/EDV ratio by MRI were associated with more extended areas of left ventricular myocardial fibrosis and with poor prognosis in hypertensive patients [13,55]. In a population of newly diagnosed and never-treated hypertensive patients, the use of LVM/EDV ratio by 3D-echo was able to recognize a higher rate of hypertensive patients with left ventricular concentric remodelling in comparison to 2D-derived relative wall thickness; these patients also presented an impairment in both systolic dynamics, with GLS and stroke volume reduction, and diastolic function, with higher values of E/e’ ratio [15,54]. Hypertensive women, being more likely to develop left ventricular concentric remodelling in response to pressure overload, presented higher values of 3D-LVM/EDV ratio than men [54,56].

3D-speckle tracking derived strains provide further information. The use of 3D-speckle tracking examination has the advantage over 2D assessment of allowing the evaluation of the entire LV from a single volume of data acquired from the apical probe position, without the need of multiple views acquisition, thus substantially reducing time of acquisition and analysis. From this technique, information about GLS, global circumferential and global radial strain, left ventricular twisting and torsion are obtainable, similarly to 2D-strain evaluation [57]. In addition to these strains, 3D-speckle tracking produces info about a further strain: global area strain, which is the percentage change of the myocardium from its original dimensions, thus a combination of both longitudinal and circumferential deformation. An impairment of all those strains, including global area strain, was observed in hypertensive patients, even in very young patients and in ones at early stages of AH [57,58]. In addition, the progression towards left ventricular geometrical remodelling induced by pressure overload exacerbated left ventricular deformation impairment, strains being more reduced, in absolute value, in hypertensive patients with concomitant LVH and dilation, according to different left ventricular geometrical patterns [16,59]. The main limitation to the use of 3D-echocardiographic approach is the vendor dependent variability and the suboptimal feasibility, which is a little lower than the standard 2D-echo approach [60]. However, improvements and advancements in transducers’ technology allow, nowadays, the acquisition of the volume data set in a single heartbeat, thus overcoming some feasibility issues due to arrhythmias or incapability in breath holding.

3D-echo found application even for the evaluation of right ventricular volumes and ejection fraction. At consolidated stages of AH, 3D RV end-systolic volume and EDV were increased, while right ventricular ejection fraction resulted reduced in patients with any type of LVH; this feature was indeed particularly evident in hypertensive patients presenting left ventricular dilation associated with LVH [61].

3D-approach was also recently used to identify early left atrial morphological and functional alterations. In particular, left atrial phasic volumes were found to correlate with organ damage induced by AH and 3D-speckle tracking derived left atrial strains resulted early altered in hypertensive patients, even before left atrial dilation [62,63]. Higher cumulative SBP from early adulthood throughout middle age was associated with adverse left atrial remodelling, with higher left atrial volumes, increased reservoir, and impaired early diastolic strain rate indicating impaired conduit function. Thus, pressure overload has an early and cumulative impact on both left atrial structure and function. Alteration of 3D LA dynamics and volumes reflect the severity and chronicity of left ventricular diastolic dysfunction, being correlated to left ventricular filling pressures [64].

## MRI

Cardiac MRI supplies multiple valuable information not only about left ventricular geometry and function, but also providing tissue characterization. Indeed, MRI is considered the gold standard for the assessment and evaluation of left ventricular mass and volumes, thanks to the optimal spatial resolution and tracing of epicardial and endocardial borders [65]. MRI allows to identify different pathological left ventricular geometrical patterns possibly present in AH: left ventricular concentric remodelling, eccentric and concentric LVH associated or not with left ventricular dilation [66]; left ventricular dilation being related with the highest risk of mortality [67]. In addition, MRI is extremely useful for guiding differential diagnosis of LVH: the possibility to combine information deriving from left ventricular geometry (thus also detect asymmetrical segmental hypertrophy) with tissue characterization and derived myocardial fibrosis’ extension and localization allows the discrimination between LVH linked to hypertensive hearts and other pathological conditions, such as infiltrative and storage cardiomyopathies, hypertrophic cardiomyopathy, athlete's heart [22,68,69]. Areas of myocardial replacement fibrosis are characterized by increased extracellular volume distribution, causing delayed gadolinium wash-out and thus detection of regions of late gadolinium enhancement (LGE). In AH-induced LVH, the presence of noninfarcted LGE patterns were recognised in about 50% of the patients, most frequently involving basal and middle segments [22]. It was also demonstrated that there was a significant correlation between the extension of LGE and the degree of left ventricular diastolic dysfunction [70]. On the contrary, in AH patients, a diffuse myocardial fibrosis is often present, which is not detectable by LGE. Indeed, T1 mapping and the evaluation of extracellular volume fraction (ECV) were introduced with this aim. T1, expressing tissues’ longitudinal relaxation time, is evaluated before and after gadolinium administration: native T1 values reflect the composite signal of both myocardial cells and interstitium, while postcontrast values give information about extracellular space. The computation of ECV is derived from a formula including pre and postcontrast T1 values of the myocardium and blood pool together with haematocrit [71,72]. In the hypertensive setting, several studies demonstrated high values of ECV and native T1 in AH patients, both being associated with left ventricular mass and LVH [73–75]. In fact, examining different patterns of left ventricular geometry related to AH, patients with LVH had significantly higher values of ECV and native T1 in comparison to those with normal left ventricular mass, whose values were comparable to those of healthy controls [13,75]. In addition, elevated ECV identified by T1 mapping in hypertensive hearts resulted associated with multiple inflammation biomarkers [75].

As AH represents a well known risk factor for the development of coronary artery disease involving both epicardial coronary arteries and coronary microcirculation, stress cardiac MRI assessment with adenosine or dipyridamole can evaluate the presence of inducible ischemia. Stress MRI by first pass gadolinium during a vasodilator stressor injection can differentiate patients with small vessel disease from those with epicardial coronary stenosis, evaluating the temporal and spatial extent of the perfusion deficits. On the contrary, subendocardial LGE can also permit to localize and quantify zones of myocardial necrosis following myocardial infarction [76,77].

Morphological and functional right ventricular adaptation to systemic pressure overload mostly reflects the modifications affecting the LV. In hypertensive patients, significant positive biventricular correlations were demonstrated between indexed mass, early peak filling rate and ejection fraction by MRI. Right ventricular mass index, ventricular wall thickness and remodelling index measured by MRI were higher in hypertensive patients than in controls [78].

Similarly to CT, MRI can provide information about morphological changes of heart (e.g. left atrial dilation) and of the cardiovascular district, thus including the possibility to uncover abnormal aortic dilation, possibly related to AH, at different districts: aortic root, arch, thoracic or abdominal aorta [79], with the advantage of not using ionizing radiation and potentially high nephrotoxic contrast agents. Furthermore, MRI is a valuable method for the evaluation of carotid plaques, both for the estimation of lumen stenosis and for the assessment of plaques’ composition, also allowing detection of lipid-rich necrotic core and intraplaque haemorrhage [80]. In the hypertensive setting, the identification of those vulnerable plaques could predict the risk of cerebrovascular events [81].

Even with MRI, it is possible to investigate myocardial deformation and strains likewise speckle tracking for echocardiography. Feature-tracking, which is an optical flow method, able to detect feature in images and track them during the cardiac cycle, allows the evaluation of GLS, global circumferential and radial strains. It is a postprocessing method and one of the most recent used, having the advantage over myocardial deformations methods, such as cardiac tagging or displacement encoding with stimulated echoes, to not need additional images acquisition [82,83]. Similarly to speckle tracking echo, feature-tracking derived strains were all demonstrated to be altered in patients affected by AH, strains being more impaired in patients with LVH [84]. In patients with hypertensive heart disease MRI-derived strains were related to both left ventricular mass and ECV [85]. The negative association between GLS by feature tracking and concentric geometry evaluated by LVM/EDV ratio was also confirmed by MRI, similarly to 3D-echocardiography [86].

Feature-tracking has been used also for the investigation of left atrial function; in AH, left atrial reservoir and conduit dysfunction were early identified in patients, even before the development of LVH [87]. These parameters correlated with E/A ratio, thus with left ventricular diastolic dysfunction. Apart from left ventricular and left atrial strain imaging, info about left ventricular diastolic function can be obtained by MRI, even if standard Doppler echo is the first level and more simple way to assess it. Left atrial enlargement, using biplane area-length method, suggests elevated left ventricular filling pressure and chronic diastolic dysfunction. Similarly to echo-Doppler assessment, phase-contrast MRI allows the evaluation of transmitral flow with E and A velocities, by placing a reference plane perpendicular to mitral inflow at the mitral valve leaflet tips. In addition, the pulmonary vein flow can be measured 1 cm into the pulmonary vein ostium. In hypertensive patients a strong relationship between MRI-derived and Doppler-derived velocities was demonstrated and MRI-derived diastolic dysfunction well correlated with left ventricular invasively measured filling pressures [88]. With technological advancement, the quantification of flow was also possible with 3D spatial encoding and four-dimensional phase contrast-MRI [89].

## COMPUTED TOMOGRAPHY

ECG-gated contrast-enhanced CT is very useful for anatomical assessment. In AH, for what concern the myocardium, CT evaluation of LVH and left atrial dilation can be performed, even if echocardiography or MRI are preferred for this analysis because free of ionizing radiations [90]. Diastolic dysfunction can be also assessed by dual-source CT with the evaluation of left atrial phasic volumes and function parameters. In addition, transmitral peak velocity could be calculated by dividing peak diastolic transmitral flow by the corresponding mitral valve area and mitral septal tissue velocity computed from changes in left ventricular length per cardiac phase for the estimation of left ventricular filling pressures. These diastolic measures presented good correlation with echo assessment [91].

Similarly to MRI, diffuse myocardial fibrosis can be detected by ECV applied to CT or dual energy CT. ECV computation again is based on haematocrit and pre and postcontrast Hounsfield unit attenuation in the myocardium and blood pool. This technique was tested in a population also including hypertensive patients, with a very good correlation between ECV by CT and ECV by MRI [92]. Thus, the possibility to noninvasively analyse myocardial fibrosis by CT, even if needing validation on wider population cohorts, has the advantage to be assessed in patients for whom MRI is precluded because of claustrophobia or non-MRI compatible implants [93].

CT has an important role in the identification and evaluation of complications related to AH. The high spatial resolution makes CT the chosen imaging method for vessels analysis. AH is associated with arterial stiffness and high mechanical stress on the aortic wall, thus resulting in alteration of aortic elastic properties and dilatation, possibly involving both thoracic and abdominal aorta [94]. The dilation of abdominal aorta has prognostic implications since it was demonstrated to be related to long-term mortality [95]. CT is, in fact, used for surveillance and for guiding surgical timing and management in patients experiencing a thoracic or abdominal aortic aneurysm, not only providing optimal views for the determination of aneurysm size and extension, but also for diagnosis of acute aortic syndromes including aortic dissections and intramural aortic hematoma, also possible complications related to AH [96,97]. In addition, AH is also one of the main risk factors for the development of coronary artery disease, as it induces endothelial coronary damage and accelerates atherosclerosis and plaque generation, and ECG-gated contrast CT provides the estimation of coronary stenosis and lumen patency [98]. CT can be also assessed to obtain information about carotid plaques composition and in particular for the evaluation of calcific volume, demonstrated to be largely present in hypertensive patients [80,99].

CT is also a valuable technique for the analysis of aortic valve. Aortic stenosis and AH often coexist, as increased pressure overload could determine a mechanical stress on the aortic leaflets, with consequent endothelial damage and development of valve stenosis [100,101]. CT could be extremely helpful in aortic stenosis grading with the possibility to evaluate calcification degree by Agaston calcium score on noncontrast CT scans, particularly in those patients in whom the echocardiographic examination is suboptimal and there is a mismatch between different echocardiographic parameters for aortic stenosis estimation [102]. In this regard, paradoxical low flow low gradient aortic stenosis is a particular type of aortic valve stenosis, characterized by a discrepancy between echocardiographic mean pressure gradient and aortic valve area, often difficult to distinguish from pseudo-severe aortic stenosis by the echocardiographic evaluation alone; it was described in hypertensive hearts with left ventricular concentric geometry and small diameters and associated with unfavourable prognosis [103].

## PET-COMPUTED TOMOGRAPHY

Molecular imaging with PET-CT is emerging for its ability in detection subclinical disease in multiple settings [104–106].

Different tracers provide information about different pathological mechanisms developed in diseases, including AH. Fluorine-18-fluorodeoxyglycose (^18^F-FDG) is a glucose analogous; an increased uptake of this tracer reflects cell active metabolism and proliferation, and it is associated with inflammation [104]. Left ventricular myocardial ^18^F-FDG uptake was demonstrated to be higher in patients affected by AH, it being positively correlated to both systolic and diastolic blood pressure values [107]. In addition, AH enhances the development of arterial atherosclerosis and related inflammation [108]. ^18^F-FDG is able to detect and quantify the arterial inflammatory processes in atherosclerotic plaques [109,110]. In particular, ^18^F-FDG uptake in carotid atheroma resulted associated with a high risk of recurrent cerebrovascular events [111].

On the other hand, fluorine-18-sodium fluoride (^18^F-NaF) is able to evaluate microcalcification activity, exchanging with hydroxyl groups on exposed regions of hydroxyapatite crystals on calcification surface [112,113]. Thus, it is extremely useful to study calcification remodelling in atherosclerotic plaques and valve diseases, both possibly related to AH condition. It was demonstrated that both diastolic and mean blood pressure values were independently associated with the extent of coronary atherosclerosis as quantified by ^18^F-NaF [114]. In addition, in a population including a high percentage of hypertensive patients, carotid 18F-NaF uptake was associated with severity of ischemic cerebrovascular disease [115]. For what concerns aortic valve stenosis, both ^18^F-FDG and ^18^F-NaF uptake resulted increased in patients with aortic stenosis. Moreover, ^18^F-NaF activity showed to be progressively increased in patients with higher disease severity [116].

Rubidium-82 PET-CT allows the evaluation of myocardial perfusion reserve, characterizing the capability of vasodilation of the coronary circulation. Hypertensive patients, in particular those with resistant hypertension, were demonstrated to exhibit lower values of myocardial perfusion reserve and this parameter was related with a higher rate of cardiovascular events, demonstrating both coronary and microvascular dysfunction and the prognostic value of myocardial perfusion reserve in this setting [117].

Even if needing wider studies on hypertensive population settings, PET-CT assessments with different tracers provide promising basis for studying molecular imaging and provide possibly prognosticators of disease progression.

## CONCLUSION

**FIGURE 2:**
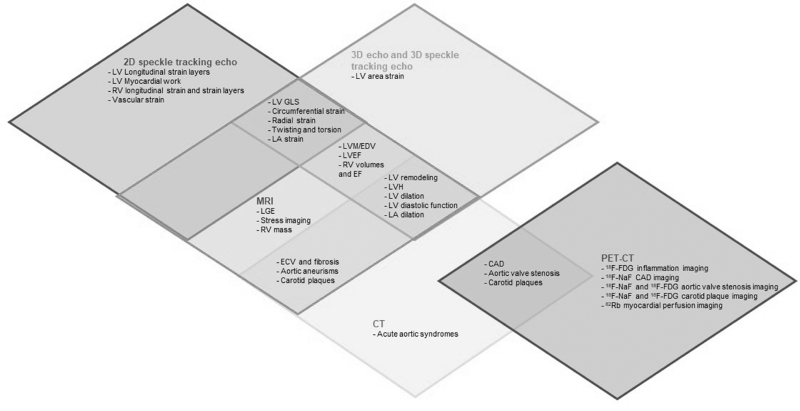
Schema depicting the advanced imaging tools useful in the cardiac and vascular damage induced by arterial hypertension; some evaluations are common to one or more techniques while each method owns its peculiarities. ^18^F-FDG, fluorine-18-fluorodeoxyglycose; ^18^F-NaF, fluorine-18-sodium fluoride; 2D, two-dimensional; 3D, three-dimensional; CAD, coronary artery disease; CT, computed tomography; ECV, extracellular volume fraction; EDV, end-diastolic volume; GLS, global longitudinal strain; LA, left atrial; LGE, late gadolinium enhancement; LV, left ventricular; LVH, left ventricular hypertrophy; LVM, left ventricular mass; PET-CT, PET-computed tomography.

The great advancements in imaging techniques allow the evaluation of AH condition and influence on the myocardium and the cardiovascular system from multiple points of view, from tools bringing to light subclinical dysfunction as speckle tracking echocardiography, to instruments allowing evaluation of tissue characterization and possible AH complication as MRI and CT, and molecular imaging highlighting active metabolism, calcification activity or microvascular dysfunction. Different imaging tools are able to detect morphological and functional abnormalities related to AH, but, on the contrary, every single technique owns its peculiarity and provides complementary information (Fig. 2).

A multiimaging approach thus may help to have a more exhaustive perspective of AH-induced damage, thus contributing to assess an early diagnosis, avoid disease progression and provide appropriate treatments for patients’ optimal management.

## ACKNOWLEDGEMENTS

M.L. is part of the PhD program in Cardiovascular Pathophysiology and Therapeutics (CardioPaTh).

### Conflicts of interest

There are no conflicts of interest.
